# Making Gold Plate and Solder

**Published:** 1852-10

**Authors:** 


### MAKING GOLD PLATE AND SOLDER.

An article on this subject, by Dr. Wood, of Nashville, Tenn., from the "Dental News Letter," which we publish in the present No. of the Register, will be useful to the Profession. It will be observed that the Dr. uses but little copper, as an alloy, and we have no doubt the small quantity with the platinum, which he combines with the silver, as an alloy, is ample with gold even twenty-two carats fine, to give all the elasticity desired.

We believe that platinum will amalgamate, or combines perfectly with gold. We have, for years, made our spiral springs with gold and platinum, and use the blow-pipe to fuse them together. But have always taken the small clippings of platinum wire.

We shall try the recipe for solder, and if the tin does not render the gold brittle, as we have found it to do heretofore, we shall regard it as a good article.

